# Altered Cytokine Levels in the First Episode of Major Depression and in Antidepressant-Naïve Patients: A Systematic Review and Meta-Analysis

**DOI:** 10.3390/ijms262110362

**Published:** 2025-10-24

**Authors:** Adam Gędek, Szymon Modrzejewski, Michał Materna, Marcin Iwański, Adam Wichniak, Monika Dominiak

**Affiliations:** 1 Third Department of Psychiatry, Institute of Psychiatry and Neurology, 02-957 Warsaw, Poland; 2 Faculty of Medicine, Medical University of Lublin, 20-059 Lublin, Poland; 3 Babinski Clinical Hospital, 30-393 Krakow, Poland; 4 Institute of Psychiatry and Neurology, 02-957 Warsaw, Poland

**Keywords:** major depression, cytokines, inflammation, first episode, drug-naïve

## Abstract

Major depressive disorder (MDD) is a severe mental disorder associated with significant functional impairment and decreased quality of life. Growing evidence suggests that immune-inflammatory mechanisms, particularly cytokine dysregulation, take part in its development and course. This systematic review and meta-analysis aimed to evaluate whether cytokine alterations are present in early stages of MDD, specifically in first-episode (FE) and drug-naïve (DN) patients. Following PRISMA guidelines a comprehensive search of PubMed, Scopus, and Web of Science was conducted in March 2025. Studies were eligible if they compared levels of inflammatory cytokines between adult FE or DN MDD patients and healthy controls (HCs). Meta-analyses using random-effects models were performed, including subanalyses depending on the source of the sample and the quality of the studies. In total, 17 eligible studies involving 1371 MDD patients were included. The meta-analysis showed significantly elevated levels of interleukin 6 (IL-6), interleukin 2 (IL-2), and tumor necrosis factor alfa (TNF-α) in FE patients compared to HCs. DN patients’ quantitative analysis showed increased levels of IL-6, IL-2, interleukin 4 (IL-4), interleukin 10 (IL-10), TNF-α, and interferon gamma (IFN-γ) compared to healthy individuals. Moreover, in the case of TNF-α, IL-2, interleukin 1 beta (IL-1β), and IL-4, there was a difference in results depending on the sample source (plasma/serum). Cytokine dysregulation is present in first-episode and drug-naïve MDD individuals. These findings highlight that the immune–inflammatory response exists in the early stages of this disorder. Moreover, since more cytokines were elevated in DN patients, pharmacological antidepressant treatment might be a significant factor involved in inflammatory regulation in MDD. Nonetheless, future prospective studies with standardized protocols and division by clinical subtypes are needed to better understand the dynamics and clinical relevance of cytokine alterations in depression.

## 1. Introduction

Major depressive disorder (MDD) is a prevalent psychiatric disorder that significantly impairs psychosocial functioning and reduces overall quality of life [1]. According to data from the World Mental Health Surveys, the estimated lifetime prevalence of MDD is 11.1%, while the annual prevalence is 5.9% [2]. Moreover, MDD ranks among the ten most disabling disorders globally [3]. The World Health Organization (WHO) projects that by 2030, MDD will become the leading contributor to the global burden of disease [4].

A substantial body of evidence has demonstrated a consistent association between depression and the activation of the inflammatory response system. It is widely hypothesized that the overexpression of pro-inflammatory cytokines contributes to the pathophysiology of depressive disorders [5,6]. Notably, interleukin-6 (IL-6), C-reactive protein (CRP), and tumor necrosis factor alpha (TNF-α) measured in the blood have been identified as the inflammatory biomarkers most consistently associated with MDD [7,8]. Moreover, a meta-analysis demonstrated elevated plasma levels of interleukin-3 (IL-3), interleukin-12 (IL-12), and interleukin-18 (IL-18) in patients with depression [9]. Cytokine level abnormalities also affect other body fluids, such as cerebrospinal fluid (CSF). The concentration of interleukin-8 (IL-8) in CSF was significantly higher in individuals with MDD compared to healthy controls (HCs) [10]. Genetic studies have also highlighted the key role of neuroinflammatory cytokines in the pathogenesis of MDD. Specifically, CRP gene methylation and polymorphisms in IL-6 (rs1800795) and interleukin-1 beta (IL-1β) (rs16944) have been identified as genetic risk loci associated with increased vulnerability to MDD [11,12,13]. Although cytokines are broadly divided into pro-inflammatory (e.g., IL-1β, IL-6, TNF-α) and anti-inflammatory (e.g., interleukin 4—IL-4, interleukin 10—IL-10, transforming growth factor β1—TGF-β1), some, including IL-6 and TGF-β1, display context-dependent activity, which may contribute to the complex and heterogeneous immune profile observed in major depressive disorder.

Chronic immune activation and elevated cytokine levels are among the most robust biological findings in depression [14]. Psychosocial stress triggers the hypothalamic–pituitary–adrenal (HPA) axis and sympathetic activation, leading to increases in glucocorticoids and catecholamines that modulate innate and adaptive immunity and promote NF-κB-mediated transcription of pro-inflammatory cytokines such as IL-6, IL-1β, and TNF-α [15]. In parallel, disturbances in the gut–brain axis—including dysbiosis and increased intestinal permeability (“leaky gut”)—facilitate the translocation of pathogen-associated molecular patterns, thereby activating Toll-like receptor signaling and systemic cytokine release [16]. These converging pathways offer biologically plausible mechanisms by which environmental stressors and host vulnerability can elevate peripheral cytokines early in major depressive disorder (MDD).

Despite these findings, several uncertainties remain regarding the relationship between inflammation and depression. MDD is a chronic condition, and it is unclear whether cytokine alterations occur immediately following disease onset or emerge over the course of its progression [17]. Moreover, pharmacological treatment may influence cytokine levels, potentially confounding the relationship between inflammation and the disorder itself. Meta-analysis suggests that levels of TNF-α and IL-8 may be modulated by antidepressant therapy [18]. In recent years, two meta-analyses evaluating cytokines in drug-naïve depression patients were conducted [19,20]. However, since then, new evidence has been published that allows for the estimation of pool effects for other compounds. In addition, the studies included in this type of meta-analysis use terminology with similar meanings—drug-naïve (patients who have never taken medication), drug-free (patients who have not taken medication at least 2 weeks), and first-episode (patients with their first diagnosis of a depressive episode). Given these complexities, the present review and meta-analysis aim to evaluate whether cytokine imbalances are significant during the early stages of MDD—specifically in individuals experiencing their first episode of depression—and whether such alterations are present in drug-naïve patients. Although numerous studies have recently focused on these populations, the findings remain inconclusive.

## 2. Materials and Methods

This systematic review was conducted according to the PRISMA statement (Preferred reporting items for systematic review and meta-analysis).

### 2.1. Data Acquisition and Search Strategy

A literature search was independently conducted by two researchers, covering studies published up to 15 March 2025, using the PubMed, Scopus, and Web of Science databases, without applying any filters. This review was guided by the following search strategy: (“depression” OR “depressive” OR “major depression” OR “major depressive disorder”) AND (“first-episode” OR “first episode” OR “drug-naïve” OR “drug naïve” OR “drug-naïve” OR “drug naive” OR “medication-naïve” OR “drug-free” OR “drug free” OR “non-treated”) AND (“interleukin” OR “cytokines” OR “cytokine” OR “tumor necrosis factor” OR “interferon” OR “transforming growth factor” OR “chemokine”). In addition, references from selected articles were screened to confirm potentially related studies.

### 2.2. Inclusion and Exclusion Criteria

The following criteria were conditions for the inclusion of the studies in this systematic review: (1) observational study design; (2) concerning DN or FE adult patients diagnosed with MDD using any recognized diagnostic criteria (e.g., DSM-5, ICD-10) and HCs with no history of mental illness; (3) at least one of the inflammatory cytokines was measured; (4) published in English. The exclusion criteria were as follows: (1) reviews, letters, and conference abstracts; (2) non-human studies; (3) duplicate data; (4) full text was not available; (5) not in English. Two investigators worked independently to complete the preliminary screening through browsing titles and abstracts. The final decisions were made after reviewing the full texts. Disagreements between the researchers were resolved by consultation with the third author.

### 2.3. Definition of Study Group

As the terminology used across studies to describe patient subgroups may vary and lead to ambiguity, we provide the definitions applied in this meta-analysis for clarity. First-episode refers to cohorts sampled during the first clinically diagnosed depressive episode, regardless of prior or current antidepressant exposure at the time of blood collection. Drug-naïve denotes cohorts in which participants had never received antidepressant treatment before sampling, regardless of the episode number. Studies explicitly reporting “first-episode, drug-naïve” were marked accordingly. In the Results section, pooled analyses are presented separately for FE and DN patient data.

### 2.4. Data Extraction

The original data was extracted from the included studies by two of the researchers. The following information was collected: the surname of the first author, year of publication, country, population, diagnosis, sample size, MDD diagnostic criteria, sample source (serum/plasma), units of measurement, cytokine levels with mean and confidence intervals or substitute statistics, matching criteria, and results regarding outcomes of interest. Any divergences were resolved through discussion between the two investigators and consultation with a third researcher.

### 2.5. Quality Assessment

The quality of the included studies was independently evaluated by two authors with the Newcastle–Ottawa Scale. The studies were assessed with respect to three aspects: Selection, Comparability, and Exposure. A maximum of one star was awarded in each category for Selection and Exposure, while a maximum of two stars could be awarded for Comparability. Studies were rated from 0 to 9, with those scoring from 0 to 2 being ranked as poor0quality, 3 to 6 as fair-quality, and 7 to 9 as high-quality. Any disagreements were managed by group discussion. High-quality and fair-quality studies were included in the meta-analysis.

### 2.6. Statistical Analysis

The meta-analysis was conducted using RevMan 5 (version 5.4.1; Cochrane Collaboration). Continuous outcomes were pooled using standardized mean differences (SMDs) with 95% confidence intervals. However, due to variability in reporting formats across studies, several methodological adjustments were applied. On account of some cytokine concentrations being reported on the logarithmic scale, all cytokine values were converted to this scale in accordance with Higgins et al. [21], to ensure consistency in effect size estimation. Additionally, for studies reporting data as interquartile ranges (IQRs), mean and standard deviation values were estimated using validated statistical conversion tools [22,23,24]. Heterogeneity was assessed both visually using forest plots and statistically using the Chi^2^ test, I^2^ statistic, and Tau^2^. Where substantial heterogeneity was detected, sensitivity analyses were performed by excluding studies identified as primary contributors to heterogeneity. Thresholds from Cochrane Collaboration were consistent with the interpretation of heterogeneity: 0–40% may not be important; 30–60% may represent moderate heterogeneity; 50–90% may represent substantial heterogeneity; and 75–100% may represent a high level of heterogeneity. A random-effect model was used for analysis, and *p* < 0.05 was set as statistical significance. Subanalyses were also conducted, taking into account high-quality studies and dividing them according to the sources of samples (serum/plasma). No other subanalyses were permitted due to the available data. Outlier exclusion was conducted as part of the sensitivity analysis for each cytokine to evaluate the influence of individual studies on the pooled effect size. Outlier exclusion was conducted as part of the sensitivity analysis for each cytokine to evaluate the influence of individual studies on the pooled effect size.

## 3. Results

### 3.1. Study Selection

**Figure 1:**
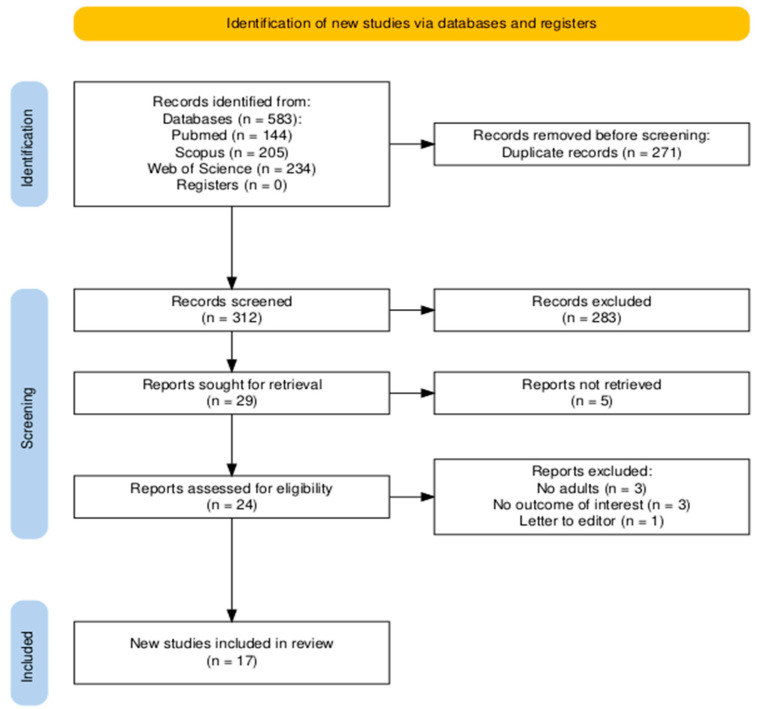
PRISMA flow diagram.

The search of three databases (PubMed, Scopus, and Web of Science) yielded a total of 583 records. After removing duplicates (*n* = 271) and screening titles and abstracts, 24 articles were selected for full-text review. Following a detailed eligibility assessment, 7 studies were excluded, resulting in 17 publications being included in the final systematic review and meta-analysis. All of the articles were included in the qualitative and quantitative analysis. The study selection process is illustrated in a PRISMA flow diagram (Figure 1) [25].

### 3.2. Characteristics of Included Studies

A total of 17 studies were included in this review. Five studies examined the FE depression patients (*n* = 5) [26,27,28,29,30], nine examined DN MDD patients (*n* = 9) [31,32,33,34,35,36,37,38,39], and three examined first-episode drug-naïve depression patients (*n* = 3) [40,41,42]. The studies included, in total, 1371 MDD patients and 861 controls. Eight of the studies were conducted in China (*n* = 8) [27,28,30,35,36,37,38,41], two each in Turkey (*n* = 2) [26,29], India (*n* = 2) [39,40], and Iraq (*n* = 2) [32,33], and one each in Taiwan (*n* = 1) [42], Korea (*n* = 1) [34], and Bangladesh (*n* = 1) [31]. Ten of the studies conducted the measurements on serum (*n* = 10) [27,28,29,30,31,32,33,34,38,40], six conducted them on plasma (*n* = 6) [26,35,37,38,41,42], and one only reported the material as “blood” (*n* = 1) [39]. Fourteen studies used an Enzyme-Linked Immunosorbent Assay (ELISA) as a method for cytokine measurement (*n* = 14) [26,28,29,30,31,32,33,34,35,36,37,38,40,42]. A multiplexed flow cytometric assay and electrochemical luminescence were each used in one study (*n* = 1) [27,41]. One of the studies did not report the method used (*n* = 1) [39]. The most frequently investigated cytokines were IL-6, examined in fourteen studies (*n* = 13) [27,28,29,31,32,34,35,36,37,38,39,41], TNF-α, examined in nine studies (*n* = 9) [26,29,30,34,35,36,38,39,41], and both transforming growth factor-beta1 (TGF-β1) [26,32,33,38,39] and IL-1β [32,34,38,41,42], in five studies each (*n* = 5). The characteristics of the included studies are presented in Table 1.

### 3.3. Risk of Bias

Table 2 provides quality scores for the papers, assessing risk of bias. Most studies were rated as high-quality, and three as fair-quality. The area where points were most frequently deducted was sample size, which emphasizes that the studies conducted so far have been based on small samples.

### 3.4. First Episode of Depression Versus Healthy Controls

#### 3.4.1. IL-6

Interleukin-6 was evaluated in five studies. The mean IL-6 was significantly higher in FE than in HCs in all of them (5/5, 100%).

**Figure 2:**
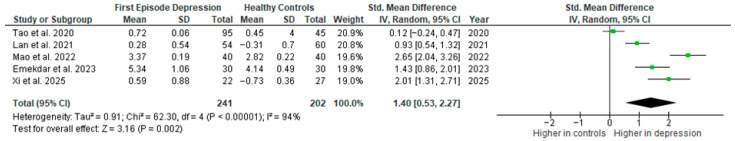
First episode of depression versus healthy controls—meta-analysis of IL-6 [27,28,29,30,41].

Results from five studies (*n* = 241/202) were included in the meta-analysis of IL-6 (Figure 2). Using the random-effect model, we did find a significantly higher level of IL-6 in FE patients than in HCs (SMD = 1.40 [0.53, 2.27], *p* = 0.002). Heterogeneity among studies was high (I^2^ = 94%). A sensitivity analysis was performed but did not result in a significant reduction in heterogeneity.

The results obtained did not differ depending on the source of the sample between serum (SMD = 1.54 [0.31, 2.76], *p* = 0.01; *n* = 4) and plasma (according to Lan et al. [41]; *n* = 1).

After including only high-quality studies in the meta-analysis, the difference remains significant (SMD = 1.40 [0.33, 2.47], *p* = 0.01; *n* = 4).

#### 3.4.2. TNF-α

Tumor necrosis factor α was evaluated in four studies. The mean TNF-α was significantly higher in FE than in HCs in all of them (4/4, 100%).

**Figure 3:**
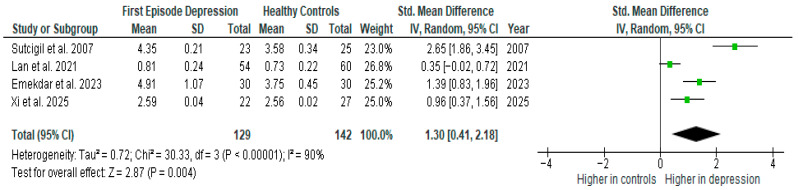
First episode of depression versus healthy controls—meta-analysis of TNF-α [26,29,30,41].

Results from four studies (*n* = 129/142) were included in the meta-analysis of TNF-α (Figure 3). Using the random-effect model, we did find a significantly higher level of TNF-α in FE patients than in HCs (SMD = 1.30 [0.41, 2.18] *p* = 0.004). Heterogeneity among studies was high (I^2^ = 90%). A sensitivity analysis was performed but did not result in a significant reduction in heterogeneity.

The results obtained differed depending on the source of the sample between serum (SMD = 1.19 [0.77, 1.61], *p* < 0.00001; *n* = 2) and plasma (SMD = 1.47 [−0.79, 3.73], *p* = 0.20; *n* = 2).

After including only high-quality studies in the meta-analysis, the difference remains significant (SMD = 1.28 [0.07, 2.49], *p* = 0.04; *n* = 3).

#### 3.4.3. IL-2

Interleukin-2 (IL-2) was evaluated in three studies. The mean IL-2 was significantly higher in FE than in HCs in all of them (3/3, 100%).

**Figure 4:**
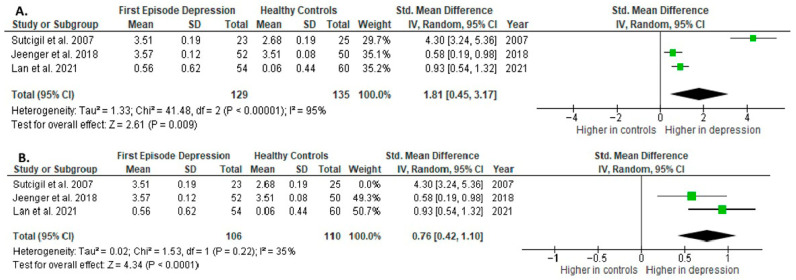
First episode of depression versus healthy controls: (**A**) meta-analysis of IL-2. (**B**) Meta-analysis of IL-2 after removing the outlier [26,40,41].

Results from three studies (*n* = 129/135) were included in the meta-analysis of IL-2 (Figure 4). Using the random-effect model, we did find a significantly higher level of IL-2 in FE patients than in HCs (SMD= 1.81 [0.45, 3.17], *p* = 0.009). Heterogeneity among studies was high (I^2^ = 95). A sensitivity analysis was performed, excluding a study identified as a primary contributor to heterogeneity. Following its removal, the heterogeneity was reduced to moderate (I^2^ = 35%), while the effect remained statistically significant (Figure 4).

The results obtained differed depending on the source of the sample between serum (SMD = 2.58 [−0.72, 5.87], *p* = 0.13; *n* = 2) and plasma (according to Jeenger et al. [40]; *n* = 1).

All studies included in the meta-analysis were high-quality.

#### 3.4.4. IL-1β

Interleukin-1β was evaluated in two studies. The mean IL-1β was significantly higher in FE than in HCs in one of them (1/2, 50%). No difference was observed in one study [35].

**Figure 5:**
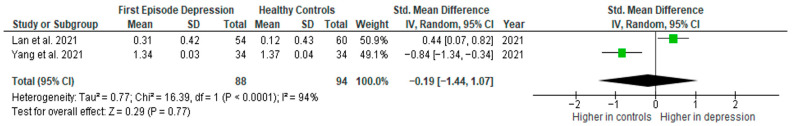
First episode of depression versus healthy controls—meta-analysis of IL-1β [41,42].

Results from two studies (*n* = 88/94) were included in the meta-analysis of IL-1β (Figure 5). Using the random-effect model, we did not find a significantly higher level of IL-1β in FE patients than in HCs (SMD= −0.19 [−1.44, 1.07], *p* = 0.77). Heterogeneity among studies was high (I^2^ = 94). Due to the limited number of studies, a sensitivity analysis could not be performed. All studies included in the meta-analysis were high-quality.

#### 3.4.5. IL-4

Interleukin-4 (IL-4) was evaluated in two studies. The mean IL-4 was not significantly higher in FE than in HCs in any of them (0/2, 0%).

**Figure 6:**
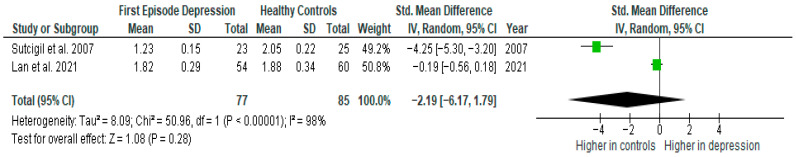
First episode of depression versus healthy controls—meta-analysis of IL-4 [26,41].

Results from two studies (*n* = 77/85) were included in the meta-analysis of IL-4 (Figure 6). Using the random-effect model, we did not find a significantly higher level of IL-4 in FE patients than in HCs (SMD= −2.19 [−6.17, 1.79], *p* = 0.28). Heterogeneity among studies was high (I^2^ = 98). Due to the limited number of studies, a sensitivity analysis could not be performed. All studies included in the meta-analysis were high-quality.

#### 3.4.6. IL-8

Interleukin-8 was evaluated in two studies. The mean IL-8 was significantly higher in FE than in HCs in one of them (1/2, 50%). No difference was observed in one study [31].

**Figure 7:**
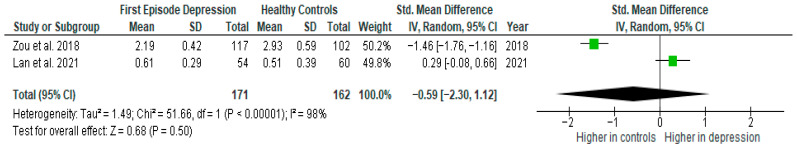
First episode of depression versus healthy controls—meta-analysis of IL-8 [38,41].

Results from two studies (*n* = 171/162) were included in the meta-analysis of IL-8 (Figure 7). Using a random-effect model, we did not find a significantly higher level of IL-8 in FE patients than in HCs (SMD= −0.59 [−2.30, 1.12], *p* = 0.50). Heterogeneity among studies was high (I^2^ = 98). Due to the limited number of studies, a sensitivity analysis could not be performed. All studies included in the meta-analysis were high-quality.

#### 3.4.7. Other Cytokines

The cytokines interleukin-5 (IL-5), interleukin-7 (IL-7), interleukin-10 (IL-10), interleukin-12, transforming growth factor beta 1 (TGF-β), interferon-gamma (IFN-γ), and interleukin-17 (IL-17) were each evaluated in a single study. Among these, the mean IL-5, IL-7 IL-10, IL-12, IFN-γ, and IL-17 were found to be significantly higher in FE patients compared to HC (1/1, 100%). The mean TGF-β1 did not show a significant difference between the FE and HC groups (0/1, 0%).

Due to the limited number of studies available for each of these cytokines, a meta-analysis could not be conducted.

### 3.5. Drug-Naïve Patients (DN) Versus Healthy Controls (HCs)

#### 3.5.1. IL-6

Interleukin-6 was evaluated in nine studies. The mean IL-6 was significantly higher in DN patients than in HCs in five of them (5/9, 56%). No difference was observed in four studies [27,28,29,31].

**Figure 8:**
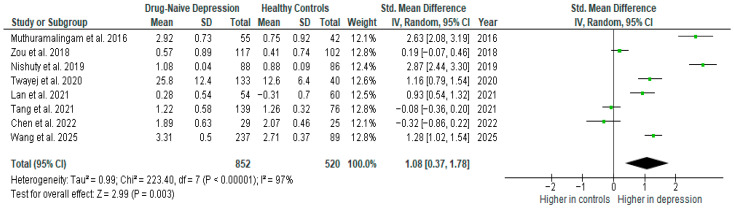
Drug-naïve patients versus healthy controls—meta-analysis of IL-6 [10,31,32,35,36,38,39,41].

Results from eight studies (*n* = 852/520) were included in the meta-analysis of IL-6 (Figure 8). Using the random-effect model, we did find a significantly higher level of IL-6 in DN patients than in HCs (SMD = 1.08 [0.37, 1.78], *p* = 0.003). Heterogeneity among studies was high (I^2^ = 97). A sensitivity analysis was performed but did not result in a significant reduction in heterogeneity.

The results obtained did not differ depending on the source of the sample between serum (SMD = 1.40 [−0.10, 2.90], *p* = 0.07; *n* = 3) and plasma (SMD = 0.47 [−0.31, 1.25], *p* = 0.24; *n* = 4); however, analyzed separately, the differences were not statistically significant.

After including only high-quality studies in the meta-analysis, the difference was on the border of statistical significance (SMD = 0.79 [−0.04, 1.62], *p* = 0.06; *n* = 6).

#### 3.5.2. TNF-α

Tumor necrosis factor α was evaluated in six studies. The mean TNF-α was significantly higher in DN patients than in HCs in most of them (5/6, 83%). No difference was observed in one study [27].

**Figure 9:**
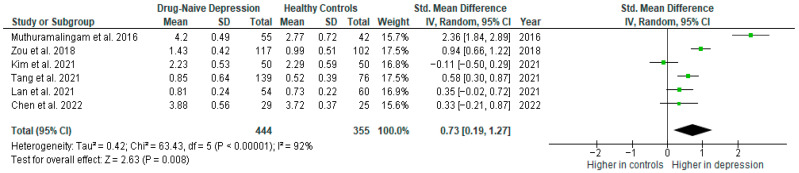
Drug-naïve patients versus healthy controls—meta-analysis of TNF-α [34,35,36,38,39,41].

Results from six studies (*n* = 444/355) were included in the meta-analysis of TNF-α (Figure 9). Using the random-effect model, we did find a significantly higher level of TNF-α in DN patients than in HCs (SMD = 0.73 [0.19, 1.27], *p* = 0.008). Heterogeneity among studies was high (I^2^ = 92). The sensitivity analysis was performed but did not result in a significant reduction in heterogeneity.

The results obtained differed depending on the source of the sample between serum (SMD = 0.43 [−0.60, 1.46], *p* = 0.41; *n* = 2) and plasma (SMD = 0.47 [0.26, 0.68], *p* < 0.0001; *n* = 3).

After including only high-quality studies in the meta-analysis, the difference remained significant (SMD = 0.44 [0.08, 0.80], *p* = 0.02; *n* = 5).

#### 3.5.3. IL-1β

Interleukin 1β was evaluated in five studies. The mean IL-1β was significantly higher in DN patients than in HCs in four of them (4/5, 80%). No difference was observed in one study [35].

**Figure 10:**
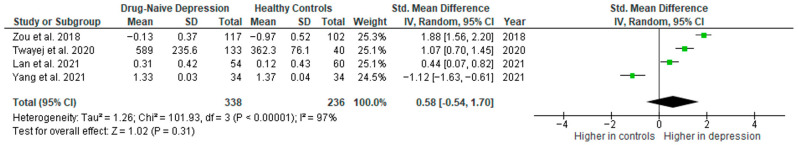
Drug-naïve patients versus healthy controls—meta-analysis of IL-1β [32,38,41,42].

Results from four studies (*n* = 338/236) were included in the meta-analysis of IL-1β (Figure 10). Using the random-effect model, we did not find a significantly higher level of IL-1β in DN patients than in HCs (SMD = 0.58 [−0.54, 1.70], *p* = 0.31). Heterogeneity among studies was high (I^2^ = 97). A sensitivity analysis was performed but did not result in a significant reduction in heterogeneity.

The results obtained differed depending on the source of the sample between serum (SMD = 1.48 [0.69, 2.27], *p* = 0.0002; *n* = 2) and plasma (SMD = −0.33 [−1.86, 1.20], *p* = 0.68; *n* = 2). All studies included in the meta-analysis were high-quality.

#### 3.5.4. TGF-β1

Transforming growth factor β1 was evaluated in four studies. The mean TGF-β1 was significantly higher in DN patients than in HCs in two of them (2/4, 80%). No difference was observed in two studies [38,39].

**Figure 11:**
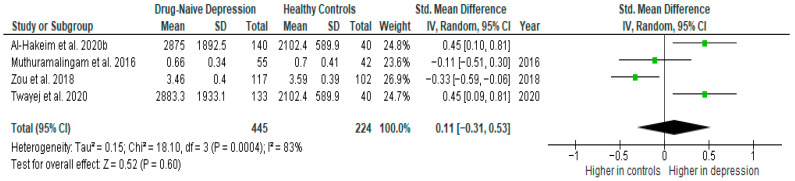
Drug-naïve patients versus healthy controls—meta-analysis of TGF-β1 [32,33,38,39].

Results from four studies (*n* = 445/224) were included in the meta-analysis of TGF-β1 (Figure 11). Using the random-effect model, we did not find a significantly higher level of TGF-β1 in DN patients than in HCs (SMD = 0.11 [−0.31, 0.53], *p* = 0.60). Heterogeneity among studies was high (I^2^ = 83). A sensitivity analysis was performed but did not result in a significant reduction in heterogeneity.

After including only high-quality studies in the meta-analysis, the difference remained not significant (SMD = 0.18 [−0.37, 0.73], *p* = 0.52; *n* = 3).

#### 3.5.5. IL-4

Interleukin 4 was evaluated in three studies. The mean IL-4 was significantly higher in DN patients than in HCs in two of them (2/3, 67%). No difference was observed in one study [41].

**Figure 12:**
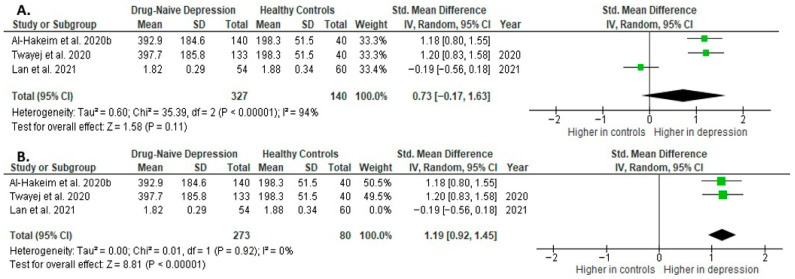
Drug-naïve patients versus healthy controls: (**A**) meta-analysis of IL-4. (**B**) Meta-analysis of IL-4 after removing the outlier [32,33,41].

Results from three studies (*n* = 327/140) were included in the meta-analysis of IL-4 (Figure 12). Using the random-effect model, we did not find a significantly higher level of IL-4 in DN patients than in HCs (SMD = 0.73 [−0.17, 1.63], *p* = 0.11). Heterogeneity among studies was high (I^2^ = 94). The sensitivity analysis was performed, excluding one study identified as a primary contributor to heterogeneity. Following its removal, heterogeneity was markedly reduced (I^2^ = 0%), and the effect became statistically significant (SMD = 1.19 [0.92, 1.45], *p* < 0.00001; *n* = 2) (Figure 12).

The results obtained differed depending on the source of the sample between serum (SMD = 1.19 [0.92, 1.45], *p* < 0.00001; *n* = 2) and plasma (according to Lan et al. [41]; *n* = 1).

After including only high-quality studies in the meta-analysis, the difference was not significant (SMD = 0.73 [−0.17, 1.63], *p* = 0.11; *n* = 3).

#### 3.5.6. IL-10

Interleukin 10 was evaluated in three studies. The mean IL-10 was significantly higher in DN patients than in HCs in all of them (3/3, 100%).

**Figure 13:**
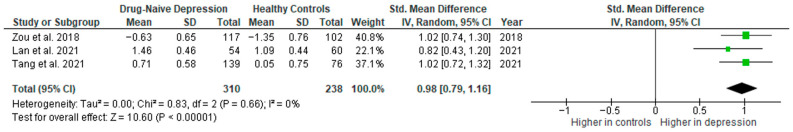
Drug-naïve patients versus healthy controls—meta-analysis of IL-10 [35,38,41].

Results from three studies (*n* = 310/238) were included in the meta-analysis of IL-10 (Figure 13). Using the random-effect model, we did find a significantly higher level of IL-10 in DN patients than in HCs (SMD = 0.98 [0.79, 1.16], *p* < 0.00001). Heterogeneity among studies was low (I^2^ = 0).

The results obtained did not differ depending on the source of the sample between serum (according to Zou et al. [38]; *n* = 1) and plasma (SMD = 0.94 [0.71, 1.18], *p* < 0.00001; *n* = 2). All studies included in the meta-analysis were high-quality.

#### 3.5.7. IFN-γ

Interferon γ was evaluated in three studies. The mean IFN-γ was significantly higher in DN patients than in HCs in all of them (3/3, 100%).

**Figure 14:**
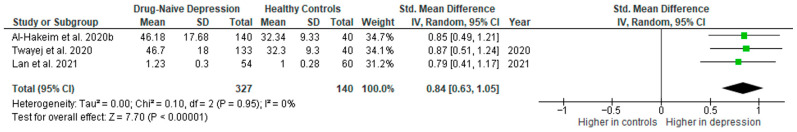
Drug-naïve patients versus healthy controls—meta-analysis of IFN-γ [32,33,41].

Results from three studies (*n* = 327/140) were included in the meta-analysis of IFN-γ (Figure 14). Using the random-effect model, we did find a significantly higher level of IFN-γ in DN patients than in HCs (SMD = 0.84 [0.63, 1.05], *p* < 0.00001). Heterogeneity among studies was low (I^2^ = 0).

The results obtained did not differ depending on the source of the sample between serum (SMD = 0.86 [0.60, 1.12], *p* < 0.00001; *n* = 2) and plasma (according to Lan et al. [41]; *n* = 1). All studies included in the meta-analysis were high-quality.

#### 3.5.8. IL-2

Interleukin 2 was evaluated by two studies. The mean IL-2 was significantly higher in DN patients than in HCs in both of them (2/2, 100%).

**Figure 15:**
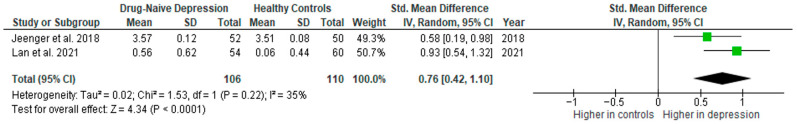
Drug-naïve patients versus healthy controls—meta-analysis of IL-2 [40,41].

Results from two studies (*n* = 106/110) were included in the meta-analysis of IL-2 (Figure 15). Using the random-effect model, we did find a significantly higher level of IL-2 in DN patients than in HCs (SMD = 0.76 [0.42, 1.10], *p* < 0.0001). Heterogeneity among studies was moderate (I^2^ = 35). Due to the limited number of studies, a sensitivity analysis could not be performed.

The results obtained did not differ depending on the source of the sample between serum (according to Jeenger et al. [40]; *n* = 1) and plasma (according to Lan et al. [41]; *n* = 1). All studies included in the meta-analysis were high-quality.

#### 3.5.9. Other Cytokines

The cytokines interleukin 5, interleukin 7, interleukin 8, interleukin 17, and interleukin 18 were each evaluated in a single study. The mean IL-5, IL-7, IL-17, and IL-18 were significantly higher in DN patients than in HCs (1/1, 100%). The mean IL-8 did not show a significant difference between the FED and HC groups (0/1, 0%).

## 4. Discussion

**Figure 16:**
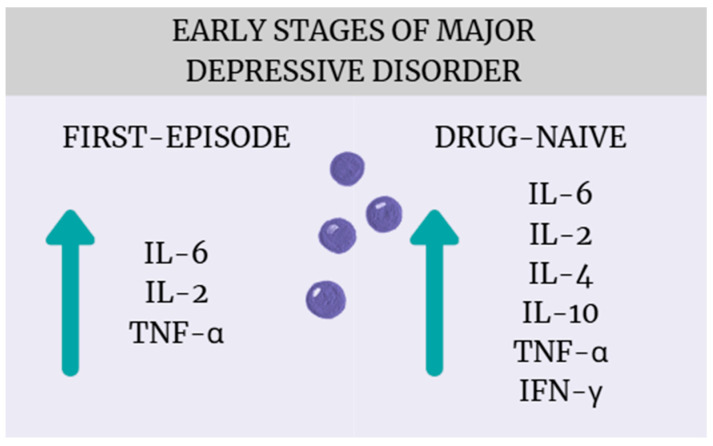
Cytokine dysregulation in the early stages of MDD.

The most important finding of this systematic review and meta-analysis is that patients with early-stage MDD, particularly those who are drug-naïve or experiencing their first episode of major depression, exhibit significant alterations in cytokine levels compared to healthy controls (Figure 16). These results align with a growing body of literature suggesting that immune–inflammatory mechanisms play an important role in the pathophysiology of MDD [43,44,45]. While many studies to date, including meta-analyses, have shown that patients with depression experience cytokine dysregulation, most of these studies include patients with many years of illness who have received numerous pharmacotherapies [8,46]. Considering that both the progression of depression and antidepressant treatment can affect inflammation in different ways, our meta-analysis provides new findings confirming that cytokine dysregulation is already present at the early stages of illness and without drug treatment. From a clinical perspective, this suggests potential diagnostic and therapeutic implications, such as the need to develop reliable biomarkers capable of identifying an “inflammatory subtype” of depression, which could facilitate patient stratification and personalized treatment approaches [47] (Miller). Moreover, the early presence of immune activation supports the potential utility of anti-inflammatory or immune-modulating interventions—either as adjunctive strategies or as preventive approaches aimed at mitigating disease progression and improving treatment response (Molero, Du) [48,49]. Notably, a broader range of cytokines was found to be elevated in drug-naïve patients (IL-6, TNF-α, IL-4, IL-2, IL-10, INF-γ) compared to those experiencing a first episode (IL-6, TNF-α, IL-2), suggesting that antidepressant treatment possibly affects inflammatory regulation.

Another important observation of this meta-analysis is the substantial heterogeneity obtained across most analyses. Although sensitivity analyses were performed, they were largely ineffective in reducing heterogeneity. While this warrants interpreting the pooled results with caution, it also reflects the considerable methodological and clinical variability among studies on depression. Despite the use of strict inclusion and exclusion criteria, the populations of patients with MDD remain highly heterogeneous in terms of illness duration, symptom profile, and comorbid conditions, which complicates the generalization of findings. Additional factors—such as differences in cytokine assessment methods, assay sensitivity, and biological sample type (serum vs. plasma)—likely contributed to the observed variability. In this meta-analysis, we observed different results depending on the source of collection for several cytokines (TNF-α, IL-2, IL-1 β, and IL-4). Furthermore, when restricted to high-quality studies, cytokine patterns differed between groups. In the FE group, results remained unchanged, while in the DN group, IL-4 became non-significant and IL-6 decreased to borderline significance. Future studies should address these issues by including larger and more homogeneous patient samples, clearly defined clinical subgroups, and standardized laboratory procedures. Comparative studies measuring cytokine levels in both serum and plasma would also be valuable to determine how the sample source influences biomarker estimates and between-study variability. While being aware of the limitations of the present analysis, we demonstrated that FE patients with MDD have higher IL-6, TNF-α, and IL-2 levels relative to HCs. In the case of DN patients with MDD, quantitative analysis showed higher IL-6, TNF-α, IL-4, IL-2, IL-10, and INF-γ levels compared to healthy individuals. These results are discussed below in the context of particular cytokines.

IL-6 was elevated in both FE (SMD = 1.40 [0.53, 2.27], *p* = 0.002) and DN (SMD = 1.08 [0.37, 1.78], *p* = 0.003) patients. Previous research has consistently implicated IL-6 in the pathophysiology of MDD [50]. Elevated IL-6 concentrations have been observed in individuals with depression [51,52,53], and some studies have additionally reported increased levels of IL-6 and its receptor antagonist in patients with treatment-resistant MDD [54,55]. Moreover, serum and plasma IL-6 levels were found to be significantly higher in FE DN individuals and medication-free MDD patients, compared to HCs [56,57], and a previous meta-analysis on the FE DN group confirmed these findings (g = 0.62; 95% CI, 0.17 to 1.06; *p* = 0.007; I^2^ = 85%) [17]. What is more, a positive correlation between serum IL-6 levels and Hamilton Depression Rating Scale (HAM-D) scores has also been documented, suggesting a link between IL-6 concentration and symptom severity in depression [51,58,59].

However, there is also evidence against the link between IL-6 and MDD. One meta-analysis has shown that IL-6 levels may decrease or remain unchanged following antidepressant treatment [60]. Another investigation including nine studies reported no significant differences in IL-6 concentrations between depressed adolescents and healthy controls [61]. Furthermore, a recent meta-analysis incorporating seven papers in the same age group did not find significantly elevated IL-6 levels in DN MDD patients compared to HCs [62]. These results indicate a probable complex contribution of IL-6 to depression, susceptible to the involvement of multiple factors such as differences in sample characteristics or the confounding effects of pharmacological interventions on cytokine expression.

The combined data revealed elevated levels of IL-2 in both FE (SMD = 1.81 [0.45, 3.17], *p* = 0.009) and DN (SMD = 0.76 [0.42, 1.10], *p* < 0.0001) patients. These findings are consistent with the previous literature, which has reported increased serum IL-2 concentrations in individuals with MDD [63]. Furthermore, one study identified an association between IL-2 levels and suicidal ideation in DN FE MDD patients, suggesting a potential link between IL-2 dysregulation and the severity or clinical features of depressive episodes [64]. Moreover, a previous meta-analysis on FE DN patients also confirmed elevated levels of this cytokine compared to HCs (g = 4.41; 95% CI, 0.13 to 8.69; *p* = 0.04; I^2^ = 98%) [20]. These findings indicate that IL-2 plays a significant role in the onset and development of depression.

FE and DN patients showed higher TNF-α levels compared to controls (SMD= 1.30 [0.41, 2.18] *p* = 0.004 and SMD = 0.73 [0.19, 1.27], *p* = 0.008, respectively). Interestingly, the previous literature presents mixed findings regarding this cytokine. For instance, one study reported significantly higher plasma TNF-α levels in medication-free FE patients compared to HCs [57]. Additionally, a previous meta-analysis confirmed elevated TNF-α concentrations in DN MDD patients relative to controls (SMD 1.04, 95% CI: 0.69–1.39, z = 5.84, *p* < 0.001) [19] and a meta-analysis on FE DN individuals also showed similar results (g = 1.21; 95% CI, 0.57 to 1.85; *p* < 0.001; I^2^ = 89%) [20]. However, other studies have found no significant differences in TNF-α levels between MDD patients and healthy individuals [46,65]. Crucially, a quantitative analysis showed that there was a difference in TNF-α concentrations regarding the source of the sample, reaching a significant difference in serum in the first episode of depression (SMD = 1.19 [0.77, 1.61], *p* < 0.00001; *n* = 2) and plasma in drug-naïve patients (SMD = 0.47 [0.26, 0.68], *p* < 0.0001; *n* = 3). These findings suggest that TNF-α might play a role in the pathology of depression, but methodological limitations and differences in sample source may hinder the recognition of this process.

The synthesized results demonstrated elevated IL-4 in DN patients (SMD = 1.19 [0.92, 1.45], *p* < 0.00001; *n* = 2). Notably, statistical significance was achieved only after excluding an outlier that contributed substantially to heterogeneity. The existing literature also provides conflicting results regarding this cytokine. A recent study reported increased IL-4 concentrations in MDD patients relative to controls, along with a positive correlation between IL-4 serum levels and HAM-D scores [66]. In contrast, Köhler et al. did not observe significant differences in IL-4 levels between MDD patients and healthy individuals [46], while Osimo et al. even observed a reduction in IL-4 concentrations among MDD patients [9]. Moreover, a previous meta-analysis failed to identify significantly elevated IL-4 levels in DN MDD patients compared to HCs [62], and a meta-analysis on FE DN individuals did not find higher levels of IL-4 in the patient group compared to HCs (g = −1.71; 95% CI, −4.73 to 1.31; *p* = 0.27; I^2^ = 97%) [20]. Importantly, in our analysis, IL-4 levels were not elevated in patients experiencing an FE (SMD = 0.73 [−0.17, 1.63], *p* = 0.11). Furthermore, we showed that IL-4 concentrations differ depending on the source—plasma/serum. These mixed findings highlight the need for further investigation into the role of IL-4 in MDD pathology, particularly in relation to treatment status and sample source.

Levels of IL-10 were higher in DN patients compared to HCs (SMD = 0.98 [0.79, 1.16], *p* < 0.00001). This finding aligns with previous research, including a recent study that reported increased serum IL-10 concentrations in drug-free MDD patients relative to HCs, along with a positive correlation between IL-10 levels and HAM-D scores [67]. Interestingly, Gazal et al. found no significant difference in IL-10 levels between MDD patients and controls [68]. However, within that study, higher IL-10 concentrations were observed in patients with later-onset depression compared to both early-onset patients and healthy individuals. In contrast, a previous meta-analysis did not find higher levels of IL-4 in FE DN patients compared to HCs (g = −0.75; 95% CI, −2.77 to 1.28; *p* = 0.47; I^2^ = 98%) [20]. Based on these results, IL-10 might be an important factor in MDD progression, but its role should be studied further, especially in terms of DN patients.

Meta-analytic integration revealed elevated levels of IFN-γ in DN patients (SMD = 0.84 [0.63, 1.05], *p* < 0.00001). Similar findings in medication-free MDD patients have been previously reported [69]; however, this is not without conflicting results. Previous meta-analysis did not identify significantly higher IFN-γ levels in DN MDD patients relative to HCs (SMD −0.05, 95% CI: −2.72–2.62, z = 0.03, *p* = 0.97) [19]. Moreover, one study reported significantly decreased serum IFN-γ levels in MDD patients compared to controls, along with a negative correlation between IFN-γ concentrations and HAM-D scores [70]. Importantly, a meta-analysis on DN FE MDD patients showed no significant rise in IFN-γ in this group (g = −0.33; 95% CI, −1.31 to 0.66; *p* = 0.51; I^2^ = 87%) [20]. These differences appear to result from the proportion of pharmacological treatment and indicate the need for further research with a better identification of the DN subgroup.

The combined data did not reveal elevated levels of IL-1β in either FE (SMD= −0.19 [−1.44, 1.07], *p* = 0.77) or DN (SMD = 0.58 [−0.54, 1.70], *p* = 0.31) patients. The findings of previous studies are contradictory. Two recent meta-analysis reported elevated levels of IL-1β in depressed individuals [9,71], suggesting a potential role in the disorder’s inflammatory profile. Furthermore, a previous meta-analysis on FE DN patients found significantly higher levels of this cytokine in depressed individuals (g = 1.52; 95% CI, 0.38 to 2.66; *p* = 0.009; I^2^ = 96%) [20]. In contrast, Haapakoski et al. did not find evidence for an association between IL-1β levels and MDD [53]. Moreover, a meta-analysis on DN MDD patients also did not find elevated IL-1β compared to HCs [62]. Importantly, this paper demonstrated difference in IL-1β levels between serum (SMD = 1.48 [0.69, 2.27], *p* = 0.0002; *n* = 2) and plasma (SMD = −0.33 [−1.86, 1.20], *p* = 0.68; *n* = 2) samples in DN individuals. This result may explain the differences observed in the above studies and indicate directions for future research.

Quantitative synthesis did not reveal higher levels of TGF-β1 in DN patients (SMD = 0.11 [−0.31, 0.53], *p* = 0.60). A previous meta-analysis also did not report significantly elevated levels of TGF-β1 in depression [9]. What is more, a meta-analysis on FE DN patients observed a trend of decreased TGF-β1 in depressed individuals (g = −0.91; 95% CI, −1.98 to 0.16; *p* = 0.10; I^2^ = 95%) [20]. Moreover, a genetic study also did not find any meaningful differences among MDD patients and healthy controls [72]. These findings suggest that TGF-β1 may not be a consistent indicator of inflammatory processes in MDD.

In addition to elevated levels of IL-6 and TNF-α in depression, these cytokines have been proven to correlate with symptoms of MDD [73]. This may suggest the potential utility of IL-6 and TNF-α as therapeutic targets. However, studies on the effect of antidepressants on these cytokines remain inconclusive [60,74]. On the one hand, future meta-analyses on large sample sizes with pre–post assessment are needed to evaluate how antidepressants affect IL-6 and TNF-α. On the other hand, anti-inflammatory treatment for mood disorders is developing [75,76], and there is evidence that anti-cytokine therapy may be effective in major depression [77]. This research indicates that it may be helpful to develop a treatment that will normalize the levels of these cytokines.

Although the precise mechanisms by which inflammatory cytokines take part in the pathogenesis of MDD remain to be fully elucidated, several biological pathways have been proposed to explain their role in the development and persistence of depressive symptoms.

Pro-inflammatory cytokines such as IL-1β and TNF-α are known to activate the hypothalamic–pituitary–adrenal axis, resulting in elevated levels of adrenocorticotropic hormone (ACTH) and cortisol [73,74,75]. This hyperactivation may impair the function of glucocorticoid receptors (GRs), thereby diminishing the anti-inflammatory effects of cortisol and perpetuating a state of chronic inflammation [76]. Notably, HPA axis dysregulation is one of the most consistently replicated biological abnormalities observed in patients with clinical depression [50,77,78]. In addition, cytokines including IFN-γ, IL-1β, IL-6, and TNF-α can induce the expression of indoleamine 2,3-dioxygenase (IDO), an enzyme that catalyzes the conversion of tryptophan into kynurenine [79,80,81,82]. This metabolic shift reduces the availability of tryptophan for serotonin (5-HT) synthesis and promotes the accumulation of neurotoxic metabolites such as quinolinic acid, which enhances glutamate release and excitotoxicity—mechanisms implicated in the neurobiology of depression [83,84]. Furthermore, elevated cytokine levels have been shown to modulate intracellular signaling pathways, leading to increased oxidative stress and neuronal apoptosis [85]. These processes may disrupt neurotransmitter signaling in key brain regions such as the prefrontal cortex and hippocampus, contributing to the emergence of affective and cognitive symptoms [86]. The activation of neuroglial cells, particularly microglia and astrocytes, by pro-inflammatory cytokines may further exacerbate neuroinflammation and synaptic dysfunction [87]. Finally, pro-inflammatory cytokines have been associated with reduced expression of brain-derived neurotrophic factor (BDNF) and its receptor—tropomyosin receptor kinase B (TrkB)—in the hippocampus [83]. This downregulation impairs neurogenesis and synaptic plasticity, both of which are critical for mood regulation and cognitive function, and may represent a key mechanism linking inflammation to the pathophysiology of MDD [88].

Antidepressant treatment may counteract these cytokine-induced neuroimmune and neuroendocrine disturbances through several complementary mechanisms. By attenuating systemic inflammation, antidepressants can normalize HPA axis function and suppress IDO activity, thereby restoring serotonergic neurotransmission and neuroplasticity [89]. In addition, several antidepressant classes, particularly selective serotonin reuptake inhibitors, have been shown to downregulate NF-κB signaling, reduce circulating concentrations of IL-6, TNF-α, and CRP, and enhance BDNF expression in the hippocampus [90]. These immunomodulatory and neurotrophic effects suggest that the therapeutic action of antidepressants extends beyond monoamine restoration, encompassing broader regulation of immune–neuroendocrine interactions implicated in the pathophysiology of major depressive disorder.

### Limitations

This study has several limitations that should be acknowledged. Firstly, substantial heterogeneity was observed in the analysis of most cytokines, likely attributable to differences in study populations, diagnostic criteria, cytokine measurement techniques, and units of quantification. Such methodological variability may have influenced the pooled effect sizes and limited the interpretability of subgroup comparisons. Secondly, for several cytokines, the number of available studies was small (often limited to two or three), which reduces the statistical power and limits the reliability and generalizability of pooled estimates. Additionally, most of the included studies were based on relatively small sample sizes, which further reduces the accuracy of subgroup analysis. Thirdly, a potential limitation of this study is the absence of multivariate analysis, which could have allowed for a more accurate estimation of the relationships between variables; however, the available data and sample size did not permit such an approach. Due to incomplete or inconsistent reporting of clinical and methodological variables across studies, it was not possible to perform additional subgroup analyses beyond sample source and study quality. Additionally, the severity of depressive symptoms was not consistently reported, preventing adjustment for this potentially important confounding factor. Fourthly, sex-related differences could not be assessed, as the majority of the included studies did not report cytokine levels separately for male and female participants. Future research should incorporate sex-stratified analyses to clarify the influence of biological sex in immune–inflammatory alterations in MDD. Another limitation is that the analyses were restricted to peripheral blood cytokines, which may not fully reflect immune processes occurring within the central nervous system. Finally, the cross-sectional nature of the included studies precludes causal inference and limits conclusions about the temporal relationship between immune alterations and the onset or progression of depression.

## 5. Conclusions

This systematic review and meta-analysis demonstrated elevated levels of inflammatory cytokines in individuals diagnosed with MDD, particularly within FE and DN subgroups, when compared to healthy controls. Specifically, increased concentrations of IL-6, IL-2, and TNF-α were observed in FE patients, while DN patients exhibited elevated levels of IL-6, IL-2, IL-4, IL-10, TNF-α, and IFN-γ. These findings reinforce the hypothesis that immune–inflammatory mechanisms, particularly cytokine-mediated pathways, play a meaningful role in the pathogenesis of MDD. Moreover, since more cytokines were elevated in DN patients, it is likely that pharmacological treatment plays a major role in inflammatory regulation. Furthermore, several methodological conclusions have also emerged. The high heterogeneity despite selectively chosen inclusion and exclusion criteria shows that populations of patients with depression can be highly heterogeneous, which should be considered in future research. Another methodological conclusion of this research is the potential difference in the value of TNF-α, IL-2, IL-1β, and IL-4 depending on the sample source—plasma or serum. These results provide direction for future prospective large-scale studies incorporating rigorous designs, harmonized measurement protocols, unified sample sources, and stratification by treatment status.

## Figures and Tables

**Table 1:** Characteristics of the included studies.

Author, Year, Country	Population, Diagnosis, Number of Patients	FE or DN	Cytokines and Sample Sources	Matched for Age and Sex	Sample Source and Units	Results
Sutcigil et al., 2007 [26], Turkey	Adults, MDD: *n* = 23 HCs: *n* = 25	FE	IL-2, IL-4, TNF-α, IL-12, TGF-β1	Yes	Plasma, pg/mL, mean	IL-4 and TGF-β1 were lower in the MDD group, whereas IL-2, IL-12, and TNF-α were higher (for all, *p* < 0.001).
Muthura-malingam et al., 2016 [39], India	Adults, MDD: *n* = 55 HCs: *n* = 42	DN	TNF-α, IL-6, TGF-β1	Yes	Blood, no info, median	TNF-α and IL-6 were significantly higher in the MDD group (for both, *p* < 0.001), while TGF-β1 was not (*p* > 0.05).
Zou et al., 2018 [38], China	Adults, MDD: *n* = 117 HCs: *n* = 102	DN	IL-1β, IL-6, IL-8, TNF-α, IL-10, TGF-β1	Yes	Serum, pg/mL, mean	IL-1β, IL-10, and TNF-α were significantly higher in the MDD group (for all, *p* < 0.01). IL-8 was significantly lower (*p* < 0.01). No difference in IL-6 or TGF-β1 was observed (for both, *p* > 0.05).
Jeenger et al., 2018 [40], India	Adults, MDD: *n* = 52 HCs: *n* = 50	FE and DN	IL-2	Yes	Serum, pg/mL, mean	IL-2 was higher in FE DN MDD patients (*p* = 0.008).
Nishuty et al., 2019 [31], Bangladesh	Adults, MDD: *n* = 88 HCs: *n* = 86	DN	IL-6	Yes	Serum, pg/mL, mean	IL-6 was significantly higher in the MDD group (*p* < 0.001).
Tao et al., 2020 [27], China	Adults, MDD: *n* = 43 HCs: *n* = 45	FE	IL-6	Yes	Serum, pg/mL, mean	IL-6 was higher in DN MDD patients (*p* = 0.027).
Twayej et al., 2020 [32], Iraq	Adults, MDD: *n* = 133 HCs: *n* = 40	DN	IL-1β, IL-4, IL-6, IL-18, IFN-γ, TGF-β1	Yes	Serum, pg/mL, mean (logarithm)	IL-1β, IL-4, IL-6, IL-18, IFN-γ, and TGFβ1 were higher in MDD patients (for all, *p* ≤ 0.001).
Al.-Hakeim et al., 2020b [33], Iraq	Adults, MDD: *n* = 140 HCs: *n* = 40	DN	IFN-γ, TGF-β1, IL-4	Yes	Serum, pg/mL, mean (logarithm)	IFN-γ, TGF-β1, and IL-4 were higher in MDD patients (for all, *p* < 0.001).
Lan et al., 2021 [41], China	Adults, MDD: *n* = 54 HCs: *n* = 60	FE and DN	IL-1β, IL-2, IL-4, IL-5, IL-6, IL-7, IL-8, IL-10, TNF-α, IFN-γ	Yes	Plasma, pg/mL, mean (logarithm)	IL-2, IL-5, IL-6, IL-7, IL-10, IFN-γ (for all, *p* < 0.001), TNF-α (*p* = 0.002), and IL-1β (*p* = 0.001) were higher in the MDD group. There was no difference in IL-4 or IL-8 (for both, *p* > 0.05).
Kim et al., 2021 [34], Korea	Adults, MDD: *n* = 50 HCs: *n* = 50	DN	IL-1β, IL-6, TNF-α, IL-17	Yes	Serum, pg/mL, mean	IL-1β and IL-17 were significantly higher in the MDD group (*p* < 0.01); (*p* = 0.0042). There was no difference for IL-6 and TNF-α (for both, *p* > 0.05).
Yang et al., 2021 [42], Taiwan	Adults, MDD: *n* = 34 HCs: *n* = 34	FE and DN	IL-1β	Yes	Plasma, pg/mL, mean	IL-1β was lower in the MDD group (*p* = 0.001).
Tang et al., 2021 [35], China	Adults, MDD: *n* = 139 HCs: *n* = 76	DN	IL-6, IL10, TNF-α	Yes	Plasma, pg/mL, mean	IL-10 and TNF-α were higher in the MDD group (for all, *p* < 0.001). There was no difference for IL-6 (*p* > 0.05).
Mao et al., 2022 [28], China	Adults, MDD: *n* = 40 HCs: *n* = 40	FE	IL-6, IL-17	Yes	Serum, pg/mL, mean	IL-6 and IL-17 were significantly higher in the MDD group (for both, *p* < 0.001).
Chen et al., 2022 [36], China	Adults, MDD: *n* = 29 HCs: *n* = 25	DN	IL-6, TNF-α	Yes	Plasma, pg/mL, mean	IL-6 and TNF-α did not differ significantly between groups (for both, *p* > 0.05).
Emekdar et al., 2023 [29], Turkey	Adults, MDD: *n* = 30 HCs: *n* = 30	FE	IL-6, TNF-α	No	Serum, ng/L, median	IL-6 was higher in the MDD group (*p* = 0.015). There was no difference in TNF-α between the groups (*p* > 0.05).
Xi et al., 2025 [30], China	Adults, MDD: *n* = 22 HCs: *n* = 27	FE	IL-6, TNF-α	Yes	Serum, pg/mL, median	IL-6 and TNF-α were higher in the MDD group (*p* = 0.008; *p* = 0.001).
Wang et al., 2025 [37], China	Adults, MDD: *n* = 237 HCs: *n* = 89	DN	IL-6	No	Plasma, pg/mL, median	IL-6 levels were significantly higher in MDD patients (*p* < 0.001).

MDD—major depressive disorder; HCs—healthy controls; FE—first episode of depression; DN—drug-naïve; IL-1β—Interleukin 1 beta; IL-2—Interleukin 2; IL-4—Interleukin 4; IL-5—Interleukin 5; IL-6—Interleukin 6; IL-7—Interleukin 7; IL-8—Interleukin 8; IL-10—Interleukin 10; IL-12—Interleukin 12; IL-17—Interleukin 17; IL-18—Interleukin 18; TNF-α—tumor necrosis factor alpha; TGF-β1—transforming growth factor beta 1; IFN-γ—Interferon gamma; pg/mL—picograms per milliliter; ng/L—nanograms per liter.

**Table 2:** Quality assessment of included studies with Newcastle–Ottawa Scale (NOS).

Studies	Selection				Comparability	Exposure			Total
	Is the Case Definition Adequate?	Representativeness of the Cases	Selection of Controls	Definition of Controls	Comparability of Cases and Controls on the Basis of the Design or Analysis	Assessment of Exposure	Same Method of Ascertainment for Cases and Controls	Non-Response Rate	
Sutcigil et al., 2007 [26], Turkey	1	1	1	1	1	1	1	0	7
Muthura-malingam et al., 2016 [39], India	1	1	1	1	1	0	1	0	6
Zou et al., 2018 [38], China	1	1	1	1	2	1	1	0	8
Jeenger et al., 2018 [40], India	1	1	1	1	1	1	1	0	7
Nishuty et al., 2019 [31], Bangladesh	1	1	1	1	2	1	1	0	8
Tao et al., 2020 [27], China	1	1	1	1	1	1	1	0	7
Twayej et al., 2020 [32], Iraq	1	1	1	1	2	1	1	0	8
Al.-Hakeim et al., 2020b [33], Iraq	1	1	1	1	2	1	1	0	8
Lan et al., 2021 [41], China	1	1	1	1	2	1	1	0	8
Kim et al., 2021 [34], Korea	1	1	1	1	2	1	1	0	8
Yang et al., 2021 [42], Taiwan	1	1	1	1	1	1	1	0	7
Tang et al., 2021 [35], China	1	1	1	1	2	1	1	0	8
Mao et al., 2022 [28], China	1	1	1	1	2	1	1	0	8
Chen et al., 2022 [36], China	1	1	1	1	2	1	1	0	8
Emekdar et al., 2023 [29], Turkey	1	1	1	1	0	1	1	0	6
Xi et al., 2025 [30], China	1	1	1	1	2	1	1	0	8
Wang et al., 2025 [37], China	1	1	1	1	0	1	1	0	6

## Data Availability

The data presented in this study are available upon request from the corresponding author. The data are not publicly available due to privacy or ethical concerns.

## Abbreviations

The following abbreviations are used in this manuscript:

5-HT	Serotonin
ACTH	Adrenocorticotropic hormone
BDNF	Brain-derived neurotrophic factor
CRP	C-reactive protein
CSF	Cerebrospinal fluid
DN	Drug-naïve
DSM-5	Diagnostic and Statistical Manual of Mental Disorders
ELISA	Enzyme-linked immunosorbent assay
FE	First-episode
GR	Glucocorticoid receptors
HAM-D	Hamilton Depression Rating Scale
HC	Healthy controls
HPA	Hypothalamic–pituitary–adrenal axis
ICD-10	International Classification of Diseases, tenth revision
IDO	Indoleamine-2,3-dioxygenase
IFN-γ	Interferon γ
IL-1β	Interleukin 1β
IL-2	Interleukin 2
IL-3	Interleukin 3
IL-4	Interleukin 4
IL-5	Interleukin 5
IL-6	Interleukin 6
IL-7	Interleukin 7
IL-8	Interleukin 8
IL-10	Interleukin 10
IL-12	Interleukin 12
IL-17	Interleukin 17
IL-18	Interleukin 18
IQR	Interquartile ranges
MDD	Major depressive disorder
NOS	Newcastle–Ottawa Scale
PRISMA	Preferred reporting items for systematic review and meta-analysis
SMD	Standardized mean difference
TGF-β1	Transforming growth factor β1
TNF-α	Tumor necrosis factor α
TrkB	Tropomyosin receptor kinase B
WHO	World Health Organization
